# Health Service Use Among Migrants in the German National Cohort—The Role of Birth Region and Language Skills

**DOI:** 10.3389/ijph.2024.1606377

**Published:** 2024-03-06

**Authors:** Christian Wiessner, Sara Licaj, Jens Klein, Barbara Bohn, Tilman Brand, Stefanie Castell, Amand Führer, Volker Harth, Margit Heier, Jana-Kristin Heise, Bernd Holleczek, Stefanie Jaskulski, Carmen Jochem, Lena Koch-Gallenkamp, Lilian Krist, Michael Leitzmann, Wolfgang Lieb, Claudia Meinke-Franze, Rafael Mikolajczyk, Ilais Moreno Velásquez, Nadia Obi, Tobias Pischon, Sabine Schipf, Sigrid Thierry, Stefan N. Willich, Hajo Zeeb, Heiko Becher

**Affiliations:** ^1^ Institute of Medical Biometry and Epidemiology, University Medical Center Hamburg-Eppendorf, Hamburg, Germany; ^2^ Institute of Medical Sociology, Center for Psychosocial Medicine, University Medical Center Hamburg-Eppendorf, Hamburg, Germany; ^3^ NAKO e.V, Heidelberg, Germany; ^4^ Leibniz Institute for Prevention Research and Epidemiology—BIPS, Bremen, Germany; ^5^ Department of Epidemiology, Helmholtz Center for Infection Research, Brunswick, Germany; ^6^ Institute of Medical Epidemiology, Biostatistics, and Informatics, Interdisciplinary Center for Health Sciences, Medical School of the Martin Luther University Halle-Wittenberg, Halle, Germany; ^7^ Institute for Occupational and Maritime Medicine Hamburg, University Medical Center Hamburg-Eppendorf, Hamburg, Germany; ^8^ Institute of Epidemiology, Helmholtz Zentrum München—German Research Center for Environmental Health (GmbH), Neuherberg, Germany; ^9^ KORA Study Centre, University Hospital Augsburg, Augsburg, Germany; ^10^ Saarland Cancer Registry, Saarbrücken, Germany; ^11^ Institute for Prevention and Cancer Epidemiology, Faculty of Medicine and Medical Center, University of Freiburg, Freiburg, Germany; ^12^ Department of Epidemiology and Preventive Medicine, University of Regensburg, Regensburg, Germany; ^13^ Department of Clinical Epidemiology and Aging Research, German Cancer Research Center (DKFZ), Heidelberg, Germany; ^14^ Institute of Social Medicine, Epidemiology and Health Economics, Charité University Medicine Berlin, Berlin, Germany; ^15^ Institute of Epidemiology, Faculty of Medicine, University of Kiel, Kiel, Germany; ^16^ Institute for Community Medicine, University Medical Center Greifswald, Greifswald, Germany; ^17^ Max-Delbrueck-Center for Molecular Medicine in the Helmholtz Association (MDC), Molecular Epidemiology Research Group, Berlin, Germany; ^18^ Max-Delbrueck-Center for Molecular Medicine in the Helmholtz Association (MDC), Biobank Technology Platform, Berlin, Germany; ^19^ Charité—Universitätsmedizin Berlin, Corporate Member of Freie Universität Berlin and Humboldt-Universität zu Berlin, Berlin, Germany; ^20^ Augsburg University Hospital, Augsburg, Germany; ^21^ Heidelberg Institute of Global Health, Heidelberg University Hospital, Heidelberg, Germany

**Keywords:** migrant health, health service research, mental health, German National Cohort, NAKO

## Abstract

**Objective:** To compare health service use (HSU) between migrants and non-migrants in Germany.

**Methods:** Using data from the population-based German National Cohort (NAKO), we compared the HSU of general practitioners, medical specialists, and psychologists/psychiatrists between six migrant groups of different origins with the utilization of non-migrants. A latent profile analysis (LPA) with a subsequent multinomial regression analysis was conducted to characterize the HSU of different groups. Additionally, separate regression models were calculated. Both analyses aimed to estimate the direct effect of migration background on HSU.

**Results:** In the LPA, the migrant groups showed no relevant differences compared to non-migrants regarding HSU. In separate analyses, general practitioners and medical specialists were used comparably to slightly more often by first-generation migrants from Eastern Europe, Turkey, and resettlers. In contrast, the use of psychologists/psychiatrists was substantially lower among those groups. Second-generation migrants and migrants from Western countries showed no differences in their HSU compared to non-migrants.

**Conclusion:** We observed a low mental HSU among specific migrant groups in Germany. This indicates the existence of barriers among those groups that need to be addressed.

## Introduction

Migrants are a large and rapidly growing group in many European countries, and they face specific challenges in navigating society, such as orientation in the healthcare system [1]. In Germany, people with migration background constitute nearly 27% of approximately 82 million inhabitants, including both first- and second-generation migrants [2]. First-generation migrants are those with foreign nationality or a foreign country of birth who migrated themselves, while second-generation migrants have at least one foreign or foreign-born parent and have no migration experience of their own.

### Health Service Use of Migrants

Although all EU member states recognize the right to the highest possible standard of physical and mental health, inequalities in health service use (HSU) between migrant and non-migrant populations exist in many European countries and may lead to adverse health outcomes [3–5]. However, general consistent patterns of HSU across countries and different migrant groups can hardly be identified, though an equitable access to health services might be related to a strong primary care system [6, 7]. In the most recent systematic review on HSU, which covered results from 10 European countries, general practitioners (GPs) were more often contacted by migrants than by non-migrants in some studies, while the opposite was observed as well [7, 8]. For medical specialists, the majority of studies indicated a lower use of outpatient specialist services by migrants compared to non-migrants. While the use of medical specialists among migrants was higher in the Nordic countries [9, 10], in Germany, the Czech Republic, Italy, and Spain a lower HSU was observed [8, 11–13]. Similarly, a recent systematic review, which included further studies published in German, found a lower use of services by medical specialists and a slightly higher use of services by GPs by migrants compared to non-migrants [4]. For preventive services, such as oral health check-ups, cancer screening, and mental health services, a consistent pattern of a lower use among migrants compared to non-migrants was observed [4, 5, 7]. For mental health services, unmet needs and lower treatment intensities were highest for persons with a recent migration experience, especially refugees [14, 15]. Conversely, migrants use emergency care more often than the non-migrant population in most European countries, which might be explained by a comparatively low knowledge of health system structures and an easier access of emergency care compared to other health services [7, 16]. This overuse of emergency care can lead to unnecessary healthcare costs that should be avoided [7, 17], and might impair long-term care because emergency services are structurally unable to function as substitutes for primary care providers.

Generally, migrants are a heterogeneous group, which makes the commonly used approach of comparing migrants with the non-migrant population without acknowledging the diversity of different migrant groups, problematic [18]. One way to operationalize the diversity of migrants is to distinguish between first- and second-generation migrants because second-generation migrants use many health services similar to the non-migrant population in Germany [11, 19–21]. A distinction between different first-generation migrants based on their country or region of origin, length of stay in the host country, and reasons for migration as well as a consideration of language proficiency and residence status may be appropriate to further reflect the heterogeneity of migrants [1, 22, 23].

### Andersen Model

The Andersen Behavioral Model of Health Service Use was used to build a theoretical framework of what influences the use of health services. According to the Andersen Model, HSU is influenced by predisposing characteristics, enabling resources, and need factors [24, 25]. Migration background is considered a predisposing characteristic because people have different preferences to use health services based on their health beliefs (e.g., attitudes, knowledge), social structure (e.g., education, occupation, and ethnicity), and demographic factors (e.g., gender, age). Enabling resources are those factors that need to be present for HSU (e.g., health insurance or the presence of health facilities nearby). Need factors represent the medical conditions that are directly related to the use of health services. An equitable access to health services would be achieved, if the HSU in a population is mainly based on its needs. For migrants, unmet needs are especially relevant regarding mental healthcare, preventive services and long-term care [5].

### Aims of the Study

As unmet needs in the provision of healthcare services might have profound negative health impacts, we aim to investigate whether migrants living in Germany differ in their propensity to use health services compared to the non-migrant population. Specifically, we want to compare different migrant groups based on their country of birth and second-generation migrants with non-migrants and investigate the influence of language skills on HSU. The health services under investigation comprise the use of services by GPs, mental health services, and different medical specialists in a period of 12 months, and are analyzed in the baseline data of a large population-based cohort study.

## Methods

### Study Population

The data stem from the German National Cohort [NAKO Gesundheitsstudie (NAKO)], a large population-based cohort study conducted at eighteen study centers in Germany since 2014. Study participants were randomly selected from population registers, resulting in a final sample size of 204,862 participants aged 19–74 years. Sufficient knowledge of the German language was required to participate. A detailed description of the study design has been published elsewhere [26]. We used questionnaire data from both a face-to-face interview and a self-administered questionnaire conducted continuously between 2014 and 2019.

### Measures of Exposure

The assignment of a migration background was based on the definition of the National Office for Statistics [2]. This classification takes into account the nationality and country of birth of both the study participants and their parents. First-generation migrants were categorized as those born without a German nationality and with a personal migration experience to Germany, while second-generation migrants were assigned a migration status if at least one parent was born without a German nationality and had a migration experience. First-generation migrants were further grouped into different regions according to their country of birth, based on the definition of the United Nations and were categorized into the following subgroups [27]:

• Western migrants (Western Europe, Northern Europe, Southern Europe, North America)
• Eastern Europe migrants
• Turkish migrants
• Resettlers (Migrants from the former Soviet Union with German ancestors)
• Other migrants (e.g., Latin America, Africa)

Additionally, non-native German speaking participants were asked about their German skills ranging from very good to very bad. Extensive data quality checks on the migration-related variables were carried out and reported elsewhere [22].

### Measures of Outcomes

**FIGURE 1:**
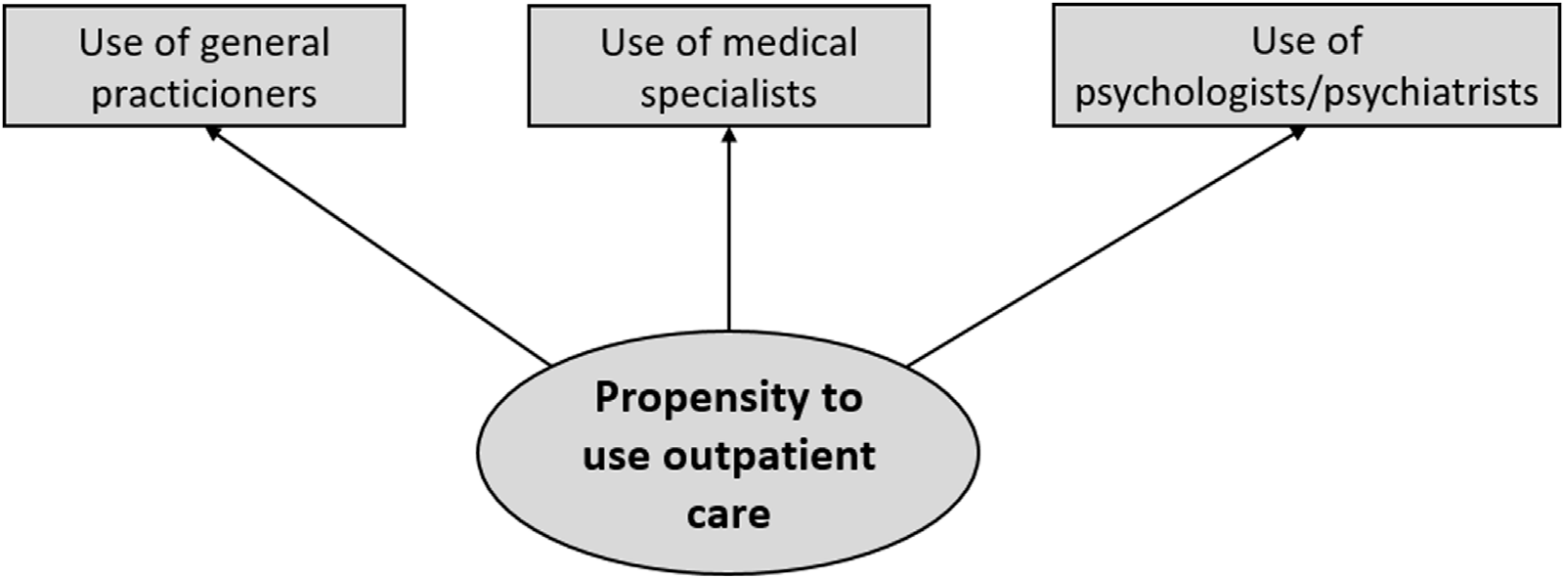
Conceptualization of the propensity to use outpatient healthcare services [German National Cohort (NAKO), Germany, 2014–2019].

HSU was measured by the use of services provided by different health professionals as reported by the study participants. A latent variable was proposed that was represented by the manifest variables of the use of services by GPs, medical specialists, and psychologists/psychiatrists (Figure 1). This latent variable, the propensity to use outpatient healthcare services, represented the outcome variable. It was evaluated by the self-reported number of visits in the past 12 months. The use of services by GPs and psychologists/psychiatrists was measured by single items, while for the medical specialists the sum of visits to internists, radiologists, neurologists, dermatologists, urologists, orthopedists, otolaryngologists, and ophthalmologists was formed.

### Measures of Covariates

As predisposing characteristics, age and sex of the participants were used. Education was categorized into low (International Standard Classification of Education (ISCED)-level 1/2), medium (ISCED-level 3/4), high (ISCED-level 5/6), and still-in-education levels based on the ISCED-97 classification [28]. Alcohol use was measured by the Alcohol Use Disorders Identification Test—Consumption (AUDIT-C), indicating the presence or absence of risky alcohol consumption. Values above 4 (men) and 3 (women) were considered a risky alcohol consumption. For the place of residence, the study center was used as a proxy. Need factors were measured by the self-reported lifetime prevalence of a medically diagnosed disease. A total of 46 diseases were assessed, and the total number of lifetime diseases was calculated for each participant. Further need factors, which we included in our analysis, were the Patient Health Questionnaire (PHQ)-9 sum score as a measure for depression and the current self-reported general health status with the values excellent, very good, good, not good, bad.

### Directed Acyclic Graph

**FIGURE 2:**
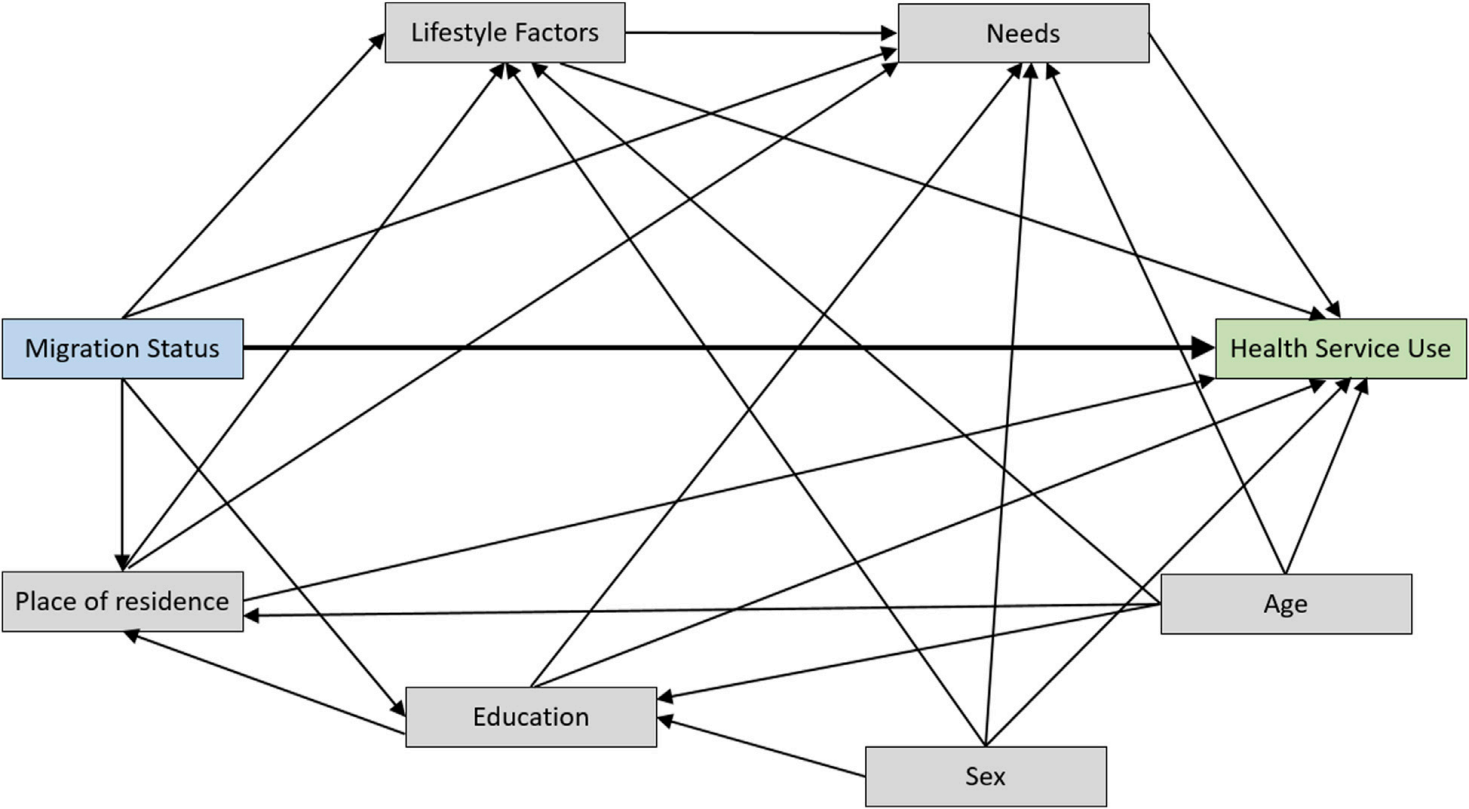
Directed acyclic graph with migration status as the exposure variable and health service use as the outcome [German National Cohort (NAKO), Germany, 2014–2019].

A directed acyclic graph (DAG) was developed to depict the assumed associations between the exposure variable (migration status), the outcome variables (HSU), and relevant covariates (Figure 2). The creation of the DAG was based on a literature search in a database for DAGs [29] and was conducted with dagitty [30]. Two DAGs with similar exposures and outcomes to the present study were found and combined with own assumptions about the underlying data-generating mechanism [31, 32]. We aimed to estimate the controlled direct effect of migration status on HSU by controlling for the effect of confounders and mediators. The minimal sufficient adjustment set (MSAS) to estimate this effect contained the following variables:

• Predisposing characteristics: Sex, Age, Education, Alcohol consumption, Place of residence (Study center)
• Need factors: Total number of diseases (lifetime), PHQ-9 sum score, General health status

### Statistical Analysis

A latent profile analysis (LPA) was conducted on participants’ responses to how often they had visited GPs, medical specialists and psychologists/psychiatrists in the previous 12 months, with the aim of identifying different patterns of HSU. A three-step procedure is most commonly used to determine the relationship between an exposure variable and the latent profiles [33]. In a first step, the number of latent profiles was determined based on model fit indices [in our case the Akaike Information Criterion (AIC) was used] and considerations about model simplicity. In a second step, we assigned people to the HSU class or pattern with the highest posterior probability of membership (modal assignment), and a putative name was derived for that class. In a third step, the exposure variable and the MSAS was included into a multinomial regression model to estimate the direct effect of migration background onto belonging to a certain latent class, and the adjusted odds ratio (aOR) with the corresponding 95% confidence interval (95% CI) was reported. In the context of HSU, a similar procedure was used by Xia et al. [34].

After the latent profile analysis, we analyzed the number of visits to GPs, medical specialists, and psychologists/psychiatrists separately in order to estimate the direct effect of migration background on these different forms of HSU. For these analyses, regression models for count data were applied with the same adjustment sets as for the latent profile analyses. The countfit function in Stata was used to compare the model fit for the different types of count regression models (Poisson regression, negative binomial regression, and the zero inflated versions of both models). For GPs and medical specialists, a negative binomial regression model provided the best model fit, while for the HSU of psychologists/psychiatrists a zero-inflated negative binomial regression model was employed because of the excess number of zeros, i.e., participants who never used the services of psychologists/psychiatrists. For the negative binomial regression models, we report the adjusted rate ratios (aRR) with the corresponding 95% CI. The inflation part of the model for the excess number of zeros is reported by the aOR with the corresponding 95% CI. In additional negative binomial regression analyses, we compared how language skills from non-native German speaking participants were associated with the HSU of GPs, medical specialists and psychologists/psychotherapists. The analyses were conducted as complete case analyses as the missingness of variables mainly occurred in the outcome variables. Along the adjusted effect estimates, we provide unadjusted effect measures. All analyses were performed in Stata (Version 17).

## Results

Of 204,862 study participants, 222 (0.1%) did not provide sufficient information for the assignment of a migration status and were removed from the analysis. The remaining study population consisted of 35,014 migrants (17.1%) and 169,626 non-migrants (82.9%) (Table 1). The majority of migrants had an own migration experience (24,563; 12.0%), while 10,451 (5.1%) participants were second-generation migrants. In total, we found migrants from 162 countries to be included into the NAKO. In the 18 study centers, the proportion of migrants ranged from 5% in a study center in Northeastern Germany to 31% in a study center in Western Germany. The study centers in Eastern Germany showed lower proportions of migrants compared to the rest of the country, with the exception of the study centers in Berlin. Most of the non-native German speaking participants reported very good (*n* = 4,957; 27.7%) or good (*n* = 7,163; 40.0%) language skills, whereas medium (*n* = 4,750; 26.5%), bad (*n* = 1,024; 5.7%) or very bad (*n* = 0; 0.0%) language skills were reported less frequently.

**TABLE 1:** Sample characteristics of different migrant groups and non-migrants [German National Cohort (NAKO), Germany, 2014–2019].

Sample characteristic^a^	Western migrants (*n* = 5,342; 2.61%)	Eastern Europe migrants (*n* = 6,992; 3.41%)	Other migrants^b^ (*n* = 5,486; 2.68%)	Turkish migrants (*n* = 3,161; 1.54%)	Resettlers (*n* = 3,582; 1.75%)	Second generation migrants (*n* = 10,451; 5.10%)	No migration background (*n* = 169,626; 82.80%)
Demographics							
Female	2,609 (49%)	4,099 (59%)	2,453 (45%)	1,352 (43%)	2,017 (56%)	5,176 (50%)	85,554 (50%)
Age, years [Mean (SD)]	50.8 (12.1)	51.0 (12.4)	46.4 (11.8)	44.7 (11.0)	46.9 (12.6)	47.9 (13.5)	50.2 (12.7)
Education							
Low	515 (11%)	232 (4%)	559 (12%)	575 (22%)	175 (5%)	237 (2%)	2,914 (2%)
Medium	1,547 (32%)	2,521 (39%)	1,358 (28%)	1,125 (43%)	1,304 (40%)	3,643 (37%)	64,896 (41%)
High	2,687 (56%)	3,561 (56%)	2,666 (56%)	831 (31%)	1,707 (52%)	5,404 (55%)	87,613 (55%)
Still in education	70 (1%)	82 (1%)	207 (4%)	115 (4%)	83 (3%)	459 (5%)	3,267 (2%)
Missing	523 (10%)	596 (9%)	696 (13%)	515 (16%)	313 (9%)	708 (7%)	10,936 (6%)
Lifestyle factors							
Risky alcohol consumption	1,503 (32%)	1,657 (26%)	786 (19%)	331 (13%)	687 (22%)	3,455 (34%)	60,825 (37%)
Missing	617 (12%)	656 (9%)	1,278 (23%)	572 (18%)	397 (11%)	354 (3%)	5,459 (3%)
Need factors							
General health status							
Not good/Bad	1,934 (40%)	1,834 (29%)	1,503 (34%)	749 (28%)	805 (25%)	3,691 (36%)	55,000 (33%)
Good	2,343 (48%)	3,710 (58%)	2,291 (52%)	1,457 (54%)	1,932 (59%)	5,309 (52%)	91,724 (56%)
Very good/Excellent	559 (12%)	886 (14%)	615 (14%)	475 (18%)	520 (16%)	1,146 (11%)	18,122 (11%)
Missing	506 (9%)	562 (8%)	1,077 (20%)	480 (15%)	325 (9%)	305 (3%)	4,780 (3%)
Lifetime diseases [Mean (SD)]	3.3 (3.0)	3.5 (3.0)	2.7 (2.7)	3.4 (3.1)	2.9 (2.7)	3.5 (2.9)	3.5 (2.9)
PHQ-9 sum score^c^ [Mean (SD)]	4.3 (4.1)	4.3 (4.0)	4.8 (4.6)	5.8 (5.1)	4.7 (4.0)	4.3 (4.0)	3.8 (3.7)
Missing	865 (16%)	1,033 (15%)	1,757 (32%)	733 (23%)	677 (19%)	571 (5%)	9,580 (6%)

^a^ Reported as *n* (%) unless specified otherwise.

^b^ Other migrants comprise migrants with origins other than Western countries, Eastern Europe, Turkey, and resettlers from the Former Soviet Union.

^c^ Patient Health Questionnaire.

In total, 148,545 (72.6%) participants completed the HSU module, which included the outcome variables for our analyses. While non-migrants (75.5% completion) and migrants of the second migration (75.1% completion) completed the questionnaire in a similar manner, migrants of the first generation showed lower completion proportions. Migrants from Western (57.8%) and Eastern European (57.7%) countries completed the module more often than migrants from other regions (Resettlers 50.4%, Turkish migrants 49.0%, Other migrants 37.9%).

In the first step of the LPA, 3 classes showed a good balance between model fit and sample sizes that allowed an investigation of migrant subgroups. In the assignment step of the LPA, the majority of the participants (108,492; 73.0%) reported a comparatively low use of GPs and medical specialists and no use of psychologists/psychiatrists. 34,644 (23.3%) participants had a medium HSU, while a small group of participants (5,409; 3.6%) reported a high HSU, especially of GPs (Table 2; Supplementary Figure S1). On average, the participants used services by GPs 2.6 times, medical specialists 3.8 times, and psychologists/psychiatrists 0.9 times in the previous 12 months. In the last step of the LPA, the low users were defined as the reference category in a multinomial regression model. After adjustment, all migrant groups were classified to each of the latent user groups with similar probabilities as the non-migrant population, i.e., all CIs for the aOR covered 1 (Table 3).

**TABLE 2:** Results of the latent profile analysis based on outpatient health service use in a period of 12 months [German National Cohort (NAKO), Germany, 2014–2019].

		Mean frequency of visits in the past 12 months (SD)
Low users (*n* = 108,492; 73.0%)	General practitioner	1.7 (1.3)
	Psychologist/psychiatrist	0.0 (0.0)
	Medical specialists	2.2 (2.0)
Medium users (*n* = 34,644; 23.3%)	General practitioner	3.6 (2.3)
	Psychologist/psychiatrist	3.2 (8.6)
	Medical specialists	7.6 (5.7)
High users (especially GPs) (*n* = 5,409; 3.6%)	General practitioner	13.5 (11.3)
	Psychologist/psychiatrist	3.5 (9.7)
	Medical specialists	10.9 (12.6)

**TABLE 3:** Multinomial regression results for the latent class outcomes based on the outpatient health service use in a period of 12 months with migration background as the exposure variable [German National Cohort (NAKO), Germany, 2014–2019].

	**Medium users^a^**				**High users (especially GPs)^a^**			
	**Unadjusted analysis**		**Adjusted analysis**		**Unadjusted analysis**		**Adjusted analysis**	
	**OR**	**95% CI**	**Adjusted OR^b^**	**95% CI**	**OR**	**95% CI**	**Adjusted OR^b^**	**95% CI**
**Second generation migrants**	1.04	0.99–1.10	1.03	0.97–1.10	1.00	0.88–1.13	0.94	0.82–1.08
**Western migrants**	1.09	1.01–1.19	1.09	0.99–1.20	1.06	0.88–1.28	1.13	0.91–1.40
**Eastern European migrants**	1.06	0.98–1.14	1.01	0.92–1.09	1.23	1.05–1.43	1.16	0.97–1.38
**Resettlers**	0.97	0.87–1.09	1.06	0.93–1.20	0.91	0.70–1.18	0.92	0.69–1.23
**Turkish migrants**	1.15	1.03–1.29	1.00	0.88–1.15	1.44	1.14–1.82	1.15	0.88–1.50
**Other migrants**	1.03	0.93–1.14	1.04	0.92–1.16	0.89	0.70–1.14	0.97	0.74–1.28

(*n* = 138,101).

^a^ Reference category: Low users.

^b^ Odds ratios are adjusted for age, sex, education, alcohol consumption, number of lifetime diseases, general health status, PHQ-9 sum score, and study center. Reference category: Non-migrants.

In separate analyses of the HSU of the different health professionals (Table 4; Supplementary Table S1), second-generation migrants and migrants from Western countries showed no difference in the use of services by GPs, medical specialists, and psychologists/psychiatrists in comparison to non-migrants. In contrast, first-generation migrants from Eastern Europe (GPs: aRR = 1.04; 95% CI 1.01–1.07; Medical specialists: aRR = 1.06; 95% CI 1.02–1.09) and resettlers (GPs: aRR = 1.05; 95% CI 1.00–1.09; Medical specialists: aRR = 1.06; 95% CI 1.01–1.11) reported a slightly higher use of services by both, GPs and medical specialists. A higher use of services by medical specialists was also reported by Turkish migrants (aRR = 1.12; 95% CI 1.07–1.19) and by a diverse group of migrants from other countries (aRR = 1.09; 95% CI 1.04–1.14). In the analysis of the use of services by psychologists/psychiatrists, only resettlers were less likely to generally be users of these services (aOR = 0.74; 95% CI 0.58–0.94; zero-inflation part of the model), while the frequency of use (count part of the model) was lower among migrants from Eastern Europe (aRR = 0.71; 95% CI 0.60–0.84), Turkey (aRR = 0.59; 95% CI 0.46–0.74), resettlers (aRR = 0.62; 95% CI 0.47–0.81), and other migrants (aRR = 0.63; 95% CI 0.50–0.79). Among non-native German speaking participants, language skills were associated with the use of psychologists/psychotherapists and less with the use of GPs and medical specialists (e.g., bad language skills vs. very good language skills: GPs: aRR = 0.95; 95% CI 0.81–1.11; Medical specialists: aRR = 0.83; 95% CI 0.70–0.99; Psychologists/psychotherapists: aRR = 0.29; 95% CI 0.11–0.74; Supplementary Table S2).

**TABLE 4:** Count regression model results for outpatient health service use in a period of 12 months with migration background as the exposure variable [German National Cohort (NAKO), Germany, 2014–2019].

	General practitioner (*n* = 138,048)		Medical specialists (*n* = 138,046)		Psychologists/psychiatrists (zero-inflation part of the model) (*n* = 137,813)		Psychologists/psychiatrists (count part of the model) (*n* = 137,813)	
	Adjusted RR^a^	95% CI	Adjusted RR^a^	95% CI	Adjusted OR^b^	95% CI	Adjusted RR^a^	95% CI
Second-generation migrants	0.99	0.97–1.01	1.01	0.99–1.03	1.00	0.90–1.11	1.07	0.95–1.20
Western migrants	1.04	1.00–1.07	1.00	0.97–1.04	1.09	0.92–1.29	1.03	0.87–1.23
Eastern European migrants	1.04	1.01–1.07	1.06	1.02–1.09	1.00	0.86–1.17	0.71	0.60–0.84
Resettlers	1.05	1.00–1.09	1.06	1.01–1.11	0.74	0.58–0.94	0.62	0.47–0.81
Turkish migrants	1.03	0.98–1.08	1.12	1.07–1.19	0.96	0.74–1.24	0.59	0.46–0.74
Other migrants	0.98	0.94–1.02	1.09	1.04–1.14	0.84	0.67–1.06	0.63	0.50–0.79

^a^ Adjusted rate ratios are adjusted for age, sex, education, alcohol consumption, number of lifetime diseases, general health status, PHQ-9 sum score, and study center. Reference category: Non-migrants.

^b^ Adjusted odds ratios with the reference categories never-users and non-migrants. Odds ratios are adjusted for age, sex, education, alcohol consumption, number of lifetime diseases, general health status, PHQ-9 sum score, and study center.

## Discussion

In this study, we aimed to compare the propensity to use health services between non-migrants and different migrant groups in the NAKO, a large population-based cohort study in Germany. We conducted two separate analyses. At first a LPA, where we could identify three different groups of health services users [low users, medium users, and high users (especially GPs)]. Thereafter, we analyzed the HSU of GPs, medical specialists, and psychologists/psychiatrists separately. In the LPA, we found no differences between the migrant groups and non-migrants. However, the separate analyses revealed a comparable to slightly higher use of services by GPs and medical specialists, and a lower use of those by psychologists/psychiatrists for different migrant groups. The most pronounced differences were found for Eastern European migrants, resettlers, Turkish migrants, and other migrants, while second-generation migrants and Western migrants used the outpatient services similar to the non-migrants.

Our results show that a general propensity to use health services could not be captured by our measures of the use of outpatient services. Markedly, the HSU was highly dependent on the specific health professional under consideration. In the LPA analysis, the slightly higher to comparable use of GPs and medical specialists among migrants was averaged out with the lower use of psychologists/psychiatrists. Therefore, the separate analyses revealed more details about the differences of HSU between migrants and non-migrants compared to the clustering-based approach in the LPA. Clustering methods such as LPA were used in several recent research papers on HSU. In a French cohort study, the use of services by GPs, medical specialists, alternative care, and emergency care was the basis for the classification [35], while Xia et al. examined the HSU of Australian truck drivers by clustering them based on their use of services by GPs, medical specialists, mental HSU, physical therapy, and surgeries [34]. These studies aimed to identify homogeneous user groups based on the actual HSU of the study population. In contrast, the QUALICOPC (Quality and Costs of Primary Care in Europe) study, a survey conducted in 34 European countries, aimed to determine the propensity to use health services through a questionnaire that asked participants about the importance of seeing a doctor for several severe hypothetical medical conditions and the expected benefit from a visit to a general practitioner for minor complaints [36]. Here, first-generation migrants reported a higher subjective importance for a doctoral visit when having severe symptoms, while a higher indicated importance was positively correlated with an actual higher HSU.

HSU due to mental health conditions is considered one of the most important priorities in the health provision for migrants [5, 37], as a migration background has been associated with a higher structural vulnerability to mental health problems [38, 39]. The discrepancy in the use of services by psychologists/psychiatrists between first- and second-generation migrants in our study can partly be explained by linguistic barriers as we found that language skills were more strongly associated with the use of services by psychologists/psychotherapists than with the use of services by GPs and medical specialists. In another study, we found that linguistic barriers and reasons related to shame were frequent causes for first-generation migrants not to seek health services related to sexual health [21]. In a recent literature review about the mental health needs of migrants, language problems were also mentioned as a key barrier to a need-based HSU [40]. Our results imply that the proportion of never-users of mental healthcare services was only higher among resettlers, while the frequency of use was substantially lower for all migrant groups except second-generation migrants and Western migrants. Aside from the aforementioned language barriers, another reason might be a lower satisfaction with the HSU, a tendency we found in a recent study as well [21].

The literature about the use of services by GPs and medical specialists among migrants in Europe provides mixed results. Both a higher and lower use of services by each type of physicians was observed in a recent European systematic review [7], while in Germany a generally lower use of services by medical specialists and a slightly higher use of GPs among migrants was found [4]. In a Norwegian register-based study, migrants used services by GPs with a lower proportion, but the users frequented their GPs more often [41]. It might be assumed that these never-users were also less likely to participate in the NAKO, which could explain the higher use of both GPs and medical specialists among the different migrant groups we investigated.

Strengths of our study were the population-based sampling design, the large sample size, which allowed the study of HSU in migrant subgroups based on their origin, and the availability of a variety of need factors and predisposing characteristics that influence HSU. However, certain limitations in our study need to be discussed. Firstly, although a population representative sampling scheme was applied in the NAKO, the response proportion was only 17% [26] and the characteristics of the study population might differ substantially from the general population, especially in the migrant group, because an inclusion criterion for the NAKO was a sufficient knowledge of the German language. Thereby, migrants with little knowledge of German as well as migrants with an irregular residence status were not part of the study. Thus, only few migrants in our study described their German language skills as bad and it can be assumed that the language skills among migrants in the general population are substantially worse than in the NAKO. Additionally, both migrants and non-migrants had comparably high levels of education, limiting the generalizability to the general German population. Secondly, the HSU module was not part of the mandatory modules in the NAKO, and only 72.6% of the participants completed it. Missingness also varied by migration status, with some migrant groups having completion proportions below 50%. Additionally, we aimed to estimate the direct effect of migration status on HSU that was not mediated through different needs, lifestyle factors, and sociodemographic characteristics. Therefore, we constructed a DAG to justify our adjustment set of variables in the regression analyses. As the true underlying data-generating mechanism is unknown to us, we might have missed further confounders and mediators. In addition to a potential selection and confounding bias, measurement bias may have occurred as well as we relied on the self-reported information about HSU and need factors. Need factors were conceptualized by the total number of lifetime diagnosed diseases, whereas the measures of HSU were based on the previous 12 months. These different time frames may have captured the need factors inaccurately, although a study by Thode et al. showed that their results did not differ substantially by including either the number of lifetime diseases or the number of diseases in the past 12 months [42]. In addition, the need factors and indicators for HSU were self-reported and not further validated.

### Conclusion

First-generation migrants from Eastern Europe, Turkey, and resettlers from the former Soviet Union all reported a comparable to slightly higher use of services by GPs and medical specialists, and a substantially lower use of those offered by psychologists/psychiatrists compared to non-migrants. In contrast, first-generation migrants from Western countries and second-generation migrants showed no differences in their HSU compared to non-migrants. These results suggest that there are unmet needs among first-generation migrants regarding the use of mental health services in Germany, which should be addressed by promoting easier access and a reduction of barriers. Due to the negative health impacts of unmet needs in the provision of healthcare services, efforts should be undertaken to tackle these barriers. These might, e.g., comprise the use of interpreters or digital tools to reduce the dependence on language skills, training of cultural competencies among healthcare workers, and the promotion of health literacy among migrants. Overall, addressing the disparities in HSU, particularly in mental healthcare, is crucial for ensuring equitable healthcare access and improving the overall wellbeing of migrant populations.
