# Supplementary material for: Health Service Use Among Migrants in the German National Cohort—The Role of Birth Region and Language Skills
Source: Int J Public Health. 2024 Mar 6;69:1606377. doi: 10.3389/ijph.2024.1606377 (PMC10952844; doi:10.3389/ijph.2024.1606377)
Supplement: Supplementary file 1 [file DataSheet1.docx]

**International Journal of Public Health**

**Health service use among migrants in the German National Cohort (NAKO) – The role of birth region and language skills**

**Figure S1.** Boxplot for the different latent profiles based on the mean number of visits to general practitioners, medical specialists and psychologists/psychiatrists in a period of 12 months


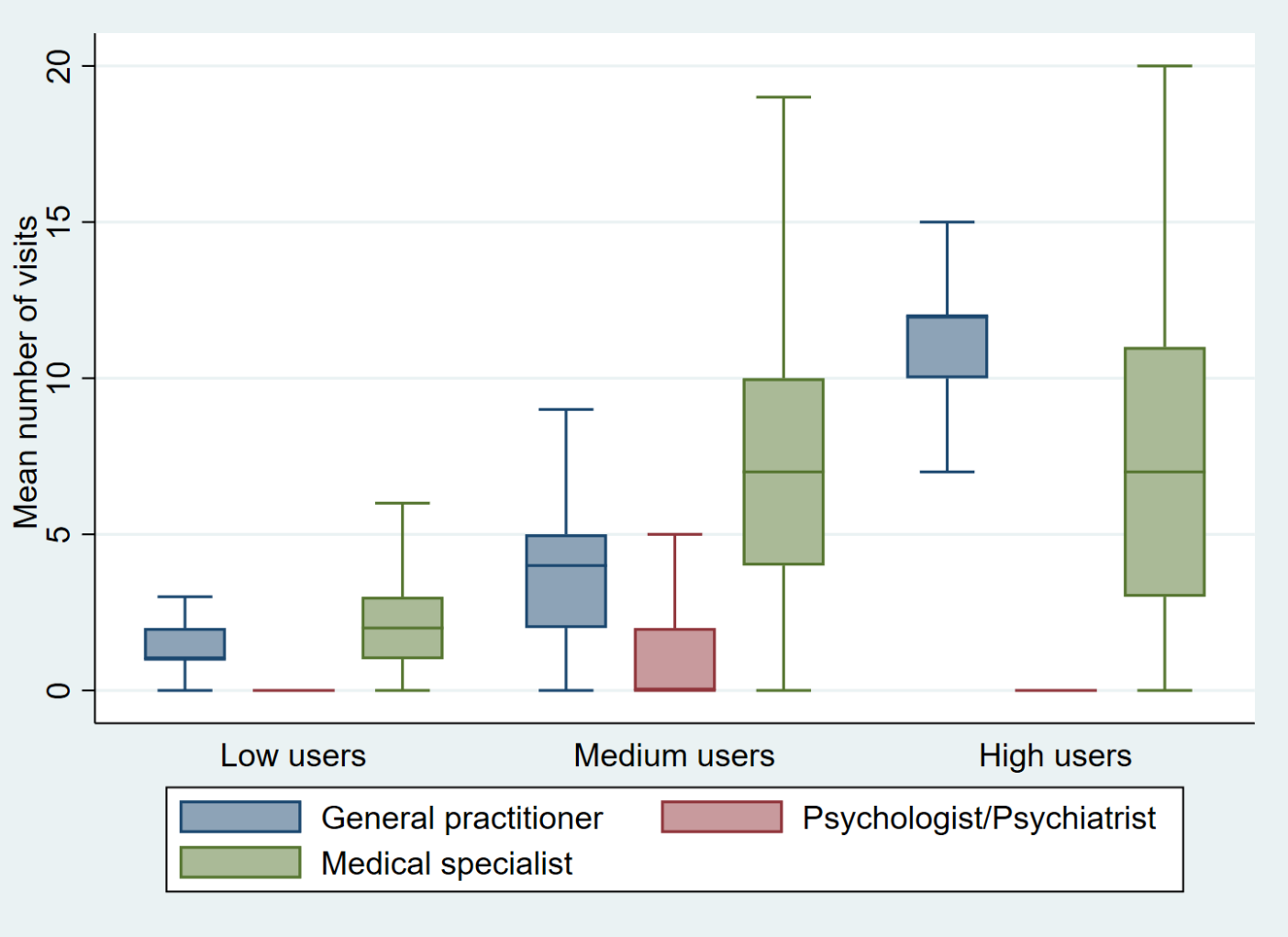


| Table S1. Unadjusted count regression model results for outpatient health service use in a period of 12 months with migration background as the exposure variable (German National Cohort (NAKO), Germany, 2014-2019). | | | | | | |  | |
| --- | --- | --- | --- | --- | --- | --- | --- | --- |
|  | General practitioner  (n=148,389) | | Medical specialists  (n=148,378) | | Psychologists/psychiatrists (Zero-inflation part of the model) (n=147,997) | | Psychologists/ psychiatrists (Count part of the model) (n=147,997) | |
|  | Unadjusted RR^1^ | 95% CI | Unadjusted RR^1^ | 95% CI | Unadjusted OR^1^ | 95% CI | Unadjusted RR^1^ | 95% CI |
| Second-generation migrants | 0.99 | 0.97-1.01 | 0.99 | 0.97-1.02 | 1.13 | 1.04-1.23 | 1.14 | 1.02-1.29 |
| Western migrants | 0.97 | 0.93-1.00 | 1.02 | 0.98-1.06 | 1.18 | 1.03-1.35 | 1.26 | 1.05-1.50 |
| Eastern European migrants | 1.04 | 1.01-1.07 | 1.10 | 1.06-1.14 | 1.17 | 1.03-1.32 | 0.75 | 0.64-0.89 |
| Resettlers | 1.01 | 0.97-1.06 | 0.97 | 0.92-1.03 | 0.93 | 0.77-1.14 | 0.76 | 0.58-0.99 |
| Turkish migrants | 1.07 | 1.02-1.12 | 1.09 | 1.03-1.15 | 1.62 | 1.34-1.96 | 0.70 | 0.56-0.88 |
| Other migrants | 0.92 | 0.88-0.96 | 1.02 | 0.98-1.07 | 1.13 | 0.95-1.35 | 0.73 | 0.58-0.92 |
| ^1^: Reference category: Non-migrants | | | | | | | | |

| Table S2. Count regression model results for outpatient health service use in a period of 12 months with language skills as the exposure variable (German National Cohort, Germany, 2014-2019). | | | | | | |
| --- | --- | --- | --- | --- | --- | --- |
| Language skills | General practitioner  (n=7,588) | | Medical specialists  (n=7,590) | | Psychologists/ psychiatrists (n=7,555) | |
|  | Adjusted RR^1^ | 95% CI | Adjusted RR^1^ | 95% CI | Adjusted RR^1^ | 95% CI |
| Good | 1.02 | 0.97–1.07 | 0.94 | 0.89–0.99 | 0.83 | 0.64–1.07 |
| Medium | 1.00 | 0.94–1.06 | 1.00 | 0.93–1.07 | 0.56 | 0.39–0.81 |
| Bad | 0.95 | 0.81–1.11 | 0.83 | 0.70–0.99 | 0.29 | 0.11–0.74 |
| ^1^: Adjusted rate ratios are adjusted for age, sex, education, alcohol consumption, number of lifetime diseases, general health status, PHQ-9 sum score, and study center. Reference category: Very good German language skills | | | | | | |
